# Spring Back Behavior of Large Multi-Feature Thin-Walled Part in Rigid-Flexible Sequential Loading Forming Process

**DOI:** 10.3390/ma15072608

**Published:** 2022-04-01

**Authors:** Yanfeng Zhang, Lihui Lang, Yao Wang, Haizhou Chen, Jianning Du, Zhihui Jiao, Lin Wang

**Affiliations:** 1 School of Mechanical Engineering and Automation, Beihang University, Beijing 100191, China; 18622709176@163.com (Y.Z.); 18740116811@163.com (Z.J.); 2 Tianjin Tianduan Press Co., Ltd., Tianjin 300142, China; chzit@126.com; 3 School of Mechanical Engineering, Hebei University of Technology, Tianjin 300130, China; 4 State Key Laboratory of Materials Processing and Die & Mould Technology, Huazhong University of Science and Technology, Wuhan 430074, China; 5 Shenyang Aircraft Industry (Group) Co., Ltd., Shenyang 110850, China; djn1221@163.com (J.D.); 18722226453@163.com (L.W.)

**Keywords:** spring back behavior, prediction model, large multi-feature thin-walled part, hydroforming, rigid-flexible sequential loading forming

## Abstract

The spring back behavior of large complex multi-feature parts in the rigid-flexible sequential forming process has certain special characteristics. The hydraulic pressure loading locus has a significant influence on the spring back of small features of the part, and the applicability of the spring back prediction model to the process needs further research. Therefore, this paper takes the large aluminum alloy inner panel of an automobile as the research object, and the spring back model and the influence laws of the hydraulic pressure loading locus are revealed by combining the theoretical analysis and numerical simulation with the process tests. Meanwhile, based on the theoretical prediction and experimental results, the spring back compensation of the complex inner panel is carried out. Results show that the hardening model has a greater impact on the accuracy of spring back prediction than the yield criterion does, and the prediction accuracy of Barlat’89 + Yoshida–Uemori mixed hardening model is the highest. Finally, the optimized loading locus of hydraulic pressure is obtained, and the accuracy results of the compensated parts verify the accuracy of the analysis model.

## 1. Introduction

The spring back problem has always been a hotspot in the field of sheet metal forming. Since the last century, scholars have carried out a large number of fruitful and effective studies on the spring back of sheet metal. The research methods include the analysis of spring back theory of typical simple shape, the establishment of spring back prediction model, and the analysis by combing numerical simulation with experiments [1,2,3]. For the spring back research of ordinary steel sheet parts such as carbon steel, stainless steel, and automobile steel, we have accumulated a wealth of experimental data and have mature research methods. However, for aluminum alloys, especially large, multi-featured, aluminum alloy, thin-walled parts, due to the complexity of deformation process and the particularity of the process, the spring back data have not accumulated in large quantities, and there is a lack of precise spring back behavior research and prediction model analysis for these kinds of material parts [4,5,6,7].

In the manufacturing of large, complex, multi-feature, thin-walled parts, the hydroforming technology has great advantages. Due to the uniform load of the high-pressure fluid, the deformation degree and uniformity of the blank are great, which can effectively reduce the spring back and improve the forming performance. However, for the precise hydroforming of complex, small, rounded corners and small features, it is necessary to load a large hydraulic pressure, which will lead to an increase in the required tonnage of the equipment. Meanwhile, it is very difficult for the part of the blank that has been molded to feed into the deformation zone. The blank in the deformation zone can only depend on the thinning of its wall thickness to adhere to the die. It is very easy to cause excessive thinning or even cracks [8,9,10]. In order to solve this problem, the authors propose a rigidflexible sequential loading forming process, which takes advantage of the dual technology of hydroforming and rigid forming, and sequentially forms the overall features and local small features of parts. The selection of the hydraulic pressure loading locus has an important influence on the spring back of the local features. Meanwhile, the accuracy of the spring back prediction results is also largely determined by the expression of the mechanical properties of the material. The anisotropy and Bauschinger effect are two important aspects that directly affect the accuracy of spring back prediction. The anisotropy of the material is closely related to the yield criterion chosen, while the Bauschinger effect is directly related to the plastic hardening model chosen [11,12,13]. Therefore, the coupling matching between the yield criterion and the plastic hardening model is of great significance for spring back prediction.

At present, more studies focus on the bending deformation of aluminum alloys and the spring back of small simple parts. There are few studies on the spring back behavior and prediction models of large, complex, multi-feature parts. Appiah et al. [14] studied the U-bending process of aluminum alloy AA6111-T4. The prediction model used was based on the Armstrong–Frederick model, and a new hybrid hardening model was proposed. The research results showed that the spring back is inversely related to the residual stress. Kinzel et al. [15] studied the spring back behavior of aluminum alloys AA6022-T4 and AA6111-T4, and verified the accuracy of the proposed spring back prediction model considering the Bauschinger effect. Tamura et al. [16] studied the spring back behavior of aluminum alloy AA5052-O and AA6016-T4 sheets, and discussed the planar anisotropy, Bauschinger effect, and cyclic hardening properties of the materials under the framework of Yoshida–Uemori hardening model and anisotropic yield criterion. Zhang et al. [17] used the Numisheet’93 U-bending test as an example to calculate the effect of different hardening models on the spring back of aluminum alloy sheets. The results showed that the spring back amount predicted by the isotropic hardening model is too large, and that predicted by the linear kinematic hardening model is small, but the proposed nonlinear mixed hardening model has high accuracy for predicting the spring back of a sheet under complex loading.

The spring back behavior of large, complex, multi-feature parts in the rigid-flexible sequential forming process has certain special characteristics. The hydraulic pressure loading locus has an important influence on the spring back of small features of the part, and the applicability of spring back prediction model to the process needs further research. Therefore, this paper takes the large aluminum alloy inner panel of an automobile as the research object, the influence laws of the hydraulic pressure loading locus and spring back model are revealed by combining the theoretical analysis and numerical simulation with the process tests. Meanwhile, based on the theoretical prediction and experimental results, the spring back compensation of the complex inner panel is carried out. The accuracy results of the compensated parts verify the accuracy of the analysis model.

## 2. Analysis of Rigid-Flexible Sequential Loading Forming Process

### 2.1. Part Material and Spring Back Characteristics

**Figure 1:**
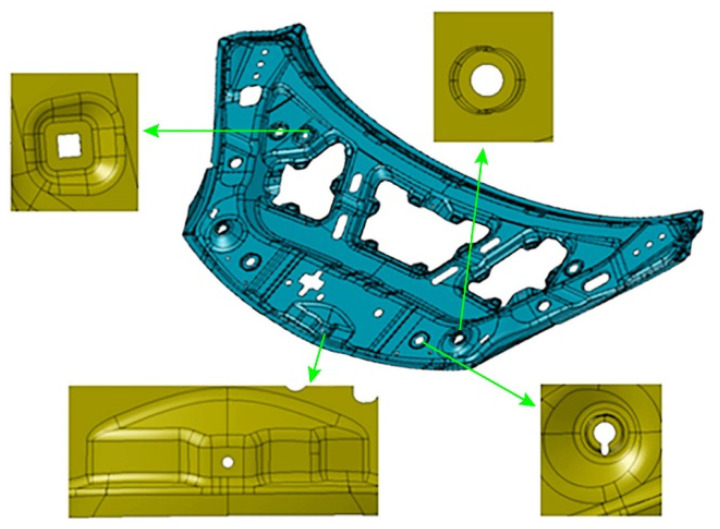
The inner panel of the engine hood and the key local features.

**Figure 2:**
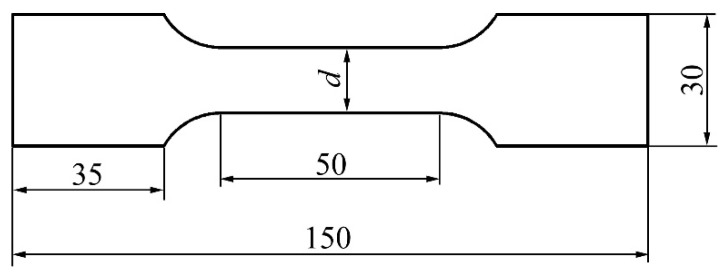
Geometry of tensile specimen.

The inner panel of the engine hood and the key local features studied in this paper are shown in Figure 1. Its shape dimensions are 1378.41 mm in length, 481.71 mm in width, and 81.05 mm in height. It has the characteristics of large overall size, small local features, and complex shape. The minimum radius of rounded corners is 2.0 mm. For the four key local features shown in Figure 1, their forming method adopted rigid-flexible sequential forming, that is, the local rigid shaping was performed by hydroforming at the mold closing position at the bottom of die. The material of the part is aluminum alloy 5182-O, the thickness is 1.0 mm, and its mechanical property was obtained by the uniaxial tensile test. The sample, with the width *D* 12.46 mm, was processed according to the shape and size of Figure 2 and relevant requirements of GB/T228.1-2010 and was selected in three directions, which are 0°, 45°, and 90°, respectively, with the rolling direction. In order to avoid the contingency of the test, two specimens were prepared for tensile test in each direction. The tensile strain rate was 0.005 s^−1^. The test results are in Table 1.

For this complex, multi-feature inner panel, the main detection positions of spring back are at the edge of contour and inner hole, which are the main positions of edge wrapping and assembly, and need to meet tolerance requirements. The edge is close to the punch nose, and the material inflow must undergo bending–reverse bending deformation, which is susceptible to cyclic hardening, and the Bausinger effect is strong. The inner hole edge also undergoes bending–reverse bending deformation in the hydroforming process, and the Bauschinger effect is obvious, the spring back can be reduced by improving the hydraulic pressure loading locus.

### 2.2. Rigid-Flexible Sequential Loading Forming

**Figure 3:**
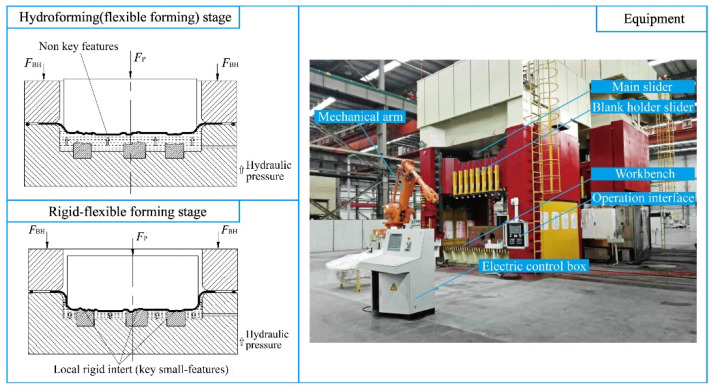
Principle of rigid-flexible sequential loading forming and equipment.

**Figure 4:**
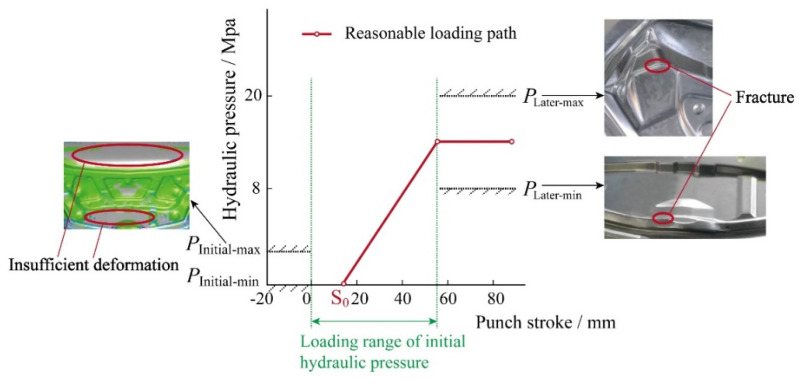
Range of hydraulic pressure loading path.

Figure 3 shows the principle of the rigid-flexible sequential loading forming process. The sheet metal undergoes hydroforming and precise clamping and shaping of local rigid inserts set at the bottom of the liquid chamber, so that the parts not formed in the hydroforming stage are formed in place, and the sequential precise forming is achieved. The spring back behavior of the part is affected by the hydraulic pressure loading locus and presents complex nonlinear characteristics. The initial hydraulic pressure should not be too large, otherwise the friction between blank and punch will keep a higher level, which is not conducive to material flow, resulting in more storage and serious spring back. Based on this, the initial hydraulic pressure should be within a range, which is represented by *P*_Initial-min_ and *P*_Initial-max_ in Figure 4. Meanwhile, the hydraulic pressure should not be prematurely loaded. If the loading is too early, the sheet will be intimately attached to the sharp features on the punch, which will result in uneven flow of the blank and a large spring back after cutting. Figure 4 shows that the hydraulic pressure begins to be loaded after the punch stroke reaches S_0_. The maximum hydraulic pressure in the later forming stage also plays an important role, too large or too small will have a negative impact on spring back. The range of maximum hydraulic pressure is expressed by *P*_Later-min_ and *P*_Later-max_ in Figure 4.

### 2.3. Spring Back Measuring Equipment and Scheme

In order to obtain the original spring back data of aluminum alloy inner panel in the rigid-flexible sequential forming, the spring back compensation was not carried out in advance. The initial values of spring back of the specimen profile were measured and obtained after forming and cutting. Based on the initial measured values and theoretical prediction results of spring back, the specimen was subjected to compensation, the mold was modified, and a new round of forming, cutting, and spring back measurement was performed. By comparison, the accuracy of the theoretical model was verified.

**Figure 5:**
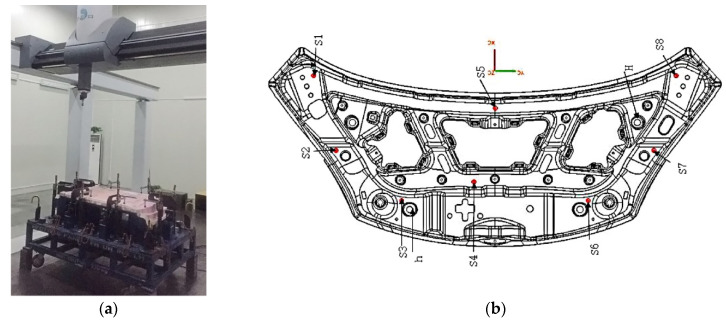
Measurement device and reference of spring back. (**a**) Three-coordinates measuring machine; (**b**) Measurement reference.

**Figure 6:**
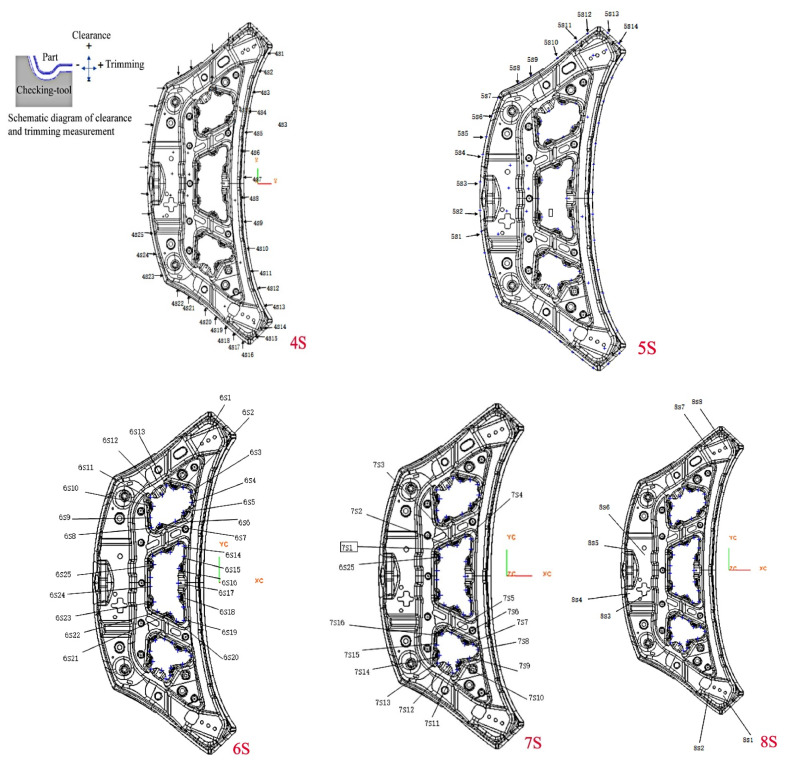
The spring back measurement positions of the profile. The 4S–8S arears are the measurement positions of spring back. Each area measures different points.

The measuring device with accuracy of 0.02 mm and reference of spring back is shown in Figure 5. The measurement value was obtained by comparing the cut profile with the checking tool profile. S1, S2, S3, S4, S5, S6, S7, and S8 are reference planes, respectively, with tolerances of 0–0.2 mm. H and h are reference hole positions, and their coordinates are H (−155, 435, 735), h (−416, −260, 659). Both reference holes have the nominal diameters of 20 mm and the diameter tolerances of 0–0.1 mm. The spring back measurement positions of the profile are shown in Figure 6, which are 5 position areas of 4S, 5S, 6S, 7S, and 8S, respectively. Twenty-five points were measured in 4S area, 14 points were measured in 5S area, 25 points were measured in 6S area, 16 points were measured in 7S area, and 8 points were measured in 8S area. Among them, the spring back tolerance of measuring points in 4S and 5S areas was ±0.5 mm, and that of measuring points in 6S, 7S, and 8S areas was ±0.7 mm. There were two specimens measured before and after spring back compensation.

## 3. Spring Back Prediction Model

The accuracy of spring back prediction results for rigid-flexible sequential forming of the large, multi-feature part depends largely on the expression of mechanical properties of materials, except for the key process parameters. The anisotropy and Bauschinger effect are two important aspects that directly affect the accuracy of spring back prediction [18,19,20,21]. The aluminum alloy sheets used in the rigid-flexible sequential forming are mostly obtained by repeated rolling and heat treatment. They have obvious fiber structure, crystalline selective orientation, and significant anisotropy. The anisotropy of materials is closely related to the yield criteria selected. Therefore, the accurate selection of yield criteria is of great significance for the numerical simulation of spring back. At present, the commonly used anisotropic yield criteria are Hill’48 anisotropic yield criterion and Barlat’89 anisotropic yield criterion.

Based on the Mises yield criterion, Hill proposed the anisotropic yield condition in 1948 [22]:

(1) $$f=\sqrt{F{\left(\sigma_{2}-\sigma_{3}\right)}^{2}+G{\left(\sigma_{3}-\sigma_{1}\right)}^{2}+H{\left(\sigma_{1}-\sigma_{2}\right)}^{2}+2L\sigma_{23}^{2}+2M\sigma_{31}^{2}+2N\sigma_{12}^{2}}=\bar{\sigma }$$

where *F*, *G*, *H*, *L*, *M*, and *N* are the anisotropic parameters of the material.

The Lankford constant is defined as follows:

(2) $$r_{0}=\frac{H}{G},r_{45}=\frac{2N-\left(F+G\right)}{2\left(F+G\right)},r_{90}=\frac{H}{F}$$

Therefore, the relationship between the anisotropy parameters and the Lankford constant is as follows:

(3) $$F=\frac{r_{0}}{\left(1+r_{0}\right)r_{90}},G=\frac{1}{1+r_{0}},H=\frac{r_{0}}{1+r_{0}},N=\frac{\left(r_{0}+r_{90}\right)\left(1+r_{45}\right)}{2r_{90}\left(1+r_{0}\right)}$$

The *L* and *M* cannot be measured, so *L* = *M* = *N* can be considered. The anisotropic parameters in Hill’48 yield function for 5182-O aluminum alloy are *F* = 0.703, *G* = 0.578, *H* = 0.422, and *N* = 1.057.

In 1989, Barlat et al. proposed a new yield criterion based on the in-plane anisotropy of materials. The yield criterion has been widely used because of its good calculation results for the stress–strain curves of aluminum alloy under biaxial tension. The equation is as follows [23]:

(4) $$\bar{\sigma }={\left\{\frac{a}{2}\left[{\left(\sigma_{1}-\sigma_{3}\right)}^{m}+{\left(-h\right)}^{m}{\left(\sigma_{2}-\sigma_{3}\right)}^{m}\right]+\left(1-\frac{a}{2}\right){\left(\sigma_{1}-\sigma_{2}-2\sigma_{3}\right)}^{m}\right\}}^{1/m}$$

where *m* is a parameter related to the crystal structure, *m* = 6 when the material is a body-centered cube, and *m* = 8 when the material is a face-centered cube. *a* and *h* are the anisotropic constants. For the 5182-O aluminum alloy, *m* = 8, *a* = 1.204, and *h* = 1.061.

The plastic hardening model is used to describe the variation of the subsequent yield function in the stress space after the material enters the plastic stage. It is related to the stress state, plastic strain, and hardening parameters of the material. For plastic reinforced materials, the commonly used hardening models are the isotropic hardening model, kinematic hardening model, and mixed hardening model.

Another important factor affecting spring back prediction accuracy of rigid-flexible sequential forming of aluminum alloy sheets is the Bauschinger effect. Especially in the forming process of complex, multi-feature inner panel, the material will repeatedly undergo forward loading, reverse loading, unloading, and other states, and the yield strength will be reduced. The Bauschinger effect is obvious. Therefore, the Bauschinger effect should be taken into account when choosing the hardening model. In the mixed hardening model, Yoshida–Uemori model is commonly used in many finite element analysis software programs. The model establishes a two-sided model for cyclic loading conditions, which introduces a boundary surface based on the yield surface, defines the isotropic hardening and kinematic hardening model in the boundary surface, and establishes the anisotropic characteristics of the material. The isotropic hardening model describes the work hardening phenomenon in the deformation process of the material, and the kinematic hardening model describes the reverse softening phenomenon, so the boundary surface is mixed hardening. The yield surface is rigidly translated in the boundary plane, which is the kinematic hardening. The model also effectively describes the Bauschinger effect [24,25].

The relative relationship between the yield surface and the boundary surface of the Yoshida–Uemori hardening model is as follow [26]:

(5) $$\alpha =\alpha^{*}-\beta$$

(6) α=B+R−Y

where α is the back stress describing the hardening of the yield surface. β is the back stress describing the hardening of boundary surface. α* is the difference between back stress α and β (MPa). *B* is the initial value of boundary surface (MPa). *Y* is the initial value of yield surface (MPa). *R* is the isotropic strengthening stress.

In the Yoshida–Uemori model, the boundary surface is mixed hardening. The expressions are as follows:

(7) $$\mathrm{Isotropic}\;\mathrm{hardening}:\;\dot{R}=m\left(R_{sat}-R\right)\dot{p}$$

(8) $$\mathrm{Kinematic}\;\mathrm{hardening}:\;\dot{\beta }=m\left(\frac{2}{3}bD^{p}-\beta \dot{p}\right)$$

where *R*_sat_ is the saturation value of the isotropic strengthening stress *R* (when the strain is infinite), *m* is the material isotropic strengthening rate parameter, and *b* is the material parameter. *D^p^* and *ṗ* are both the equivalent plastic strain rate.

Yoshida revised the original model to deal with the rapid change of work hardening rate after the initial yield in most cases. The parameters *C*_1_ and *C*_2_ are defined for the difference between the forward and reverse loading. The correction is as follows [27]:

(9) $$R=R_{sat}\left[{\left(C_{1}+{\bar{\varepsilon }}^{p}\right)}^{C_{2}}-C_{1}{}^{C_{2}}\right]$$

The revised Yoshida–Uemori hardening model has nine parameters: *Y*, *B*, *C*, *R*_sat_, *b*, *m*, *h*, *C*_1_, and *C*_2_. For the 5182-O aluminum, alloy, these parameters, obtained by tests, are shown in Table 2.

The spring back prediction models were obtained by matching different yield criteria and plastic hardening models. For the large, complex, multi-feature inner panel, four spring back models were studied, including Barlat’89 + Hollomon isotropic hardening model, Hill’48 + Hollomon isotropic hardening model, Hill’48 + Yoshida–Uemori mixed hardening model, and Barlat’89 + Yoshida–Uemori mixed hardening model. Meanwhile, the applicability of spring back models to the rigid-flexible sequential loading forming process was verified.

## 4. Results and Discussion

### 4.1. Prediction Accuracy of Spring Back Model

From the perspective of plasticity theory, the complete material model should include the material flow stress relationship, the initial yield criterion, and the subsequent yield hardening model. In the simulation, the associated flow theory was used in the flow relation equation. The combination of different initial yield criteria and hardening models with the true stress–strain relationship of the material obtained by tensile tests formed different material models and different spring back prediction models. The rigid-flexible sequential forming process of aluminum alloy automobile inner panel is a complex plastic deformation process. The applicability of different spring back models to this process needs further study.

**Figure 7:**
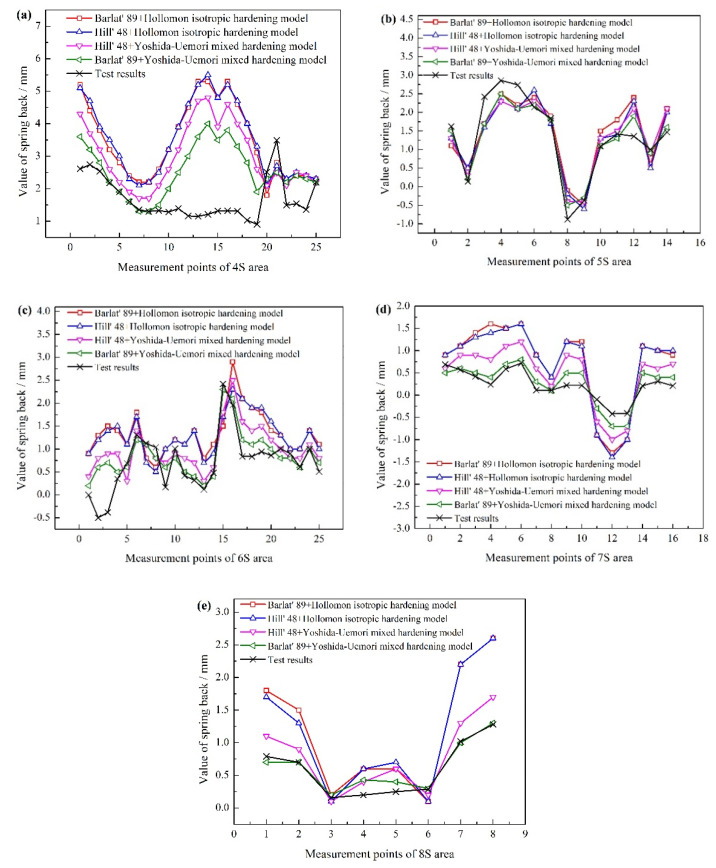
The comparisons between prediction results and the test results: (**a**) 4S area, (**b**) 5S area, (**c**) 6S area, (**d**) 7S area, and (**e**) 8S area.

The comparisons between the prediction results of the four kinds of spring back models and the test results in different measurement areas are shown in Figure 7. It can be seen that the prediction results of spring back models are larger than the test values. The prediction accuracy of Barlat’89 + Yoshida–Uemori mixed hardening model is the highest and its consistency with the test results is the best. The second is the Hill’48 + Yoshida–Uemori mixed hardening model; the prediction accuracy of the spring back model combined with Hollomon isotropic hardening model is lower. The spring back predicted by isotropic hardening model is larger than that predicted by mixed hardening model, and the difference between the values of isotropic hardening model and test is also larger. When the isotropic hardening model is adopted, the prediction accuracy of the Hill’48 yield criterion is slightly higher than that of the Barlat’89 yield criterion. When the mixed hardening model is adopted, the prediction accuracy of the Barlat’89 yield criterion is significantly higher than that of Hill’48 yield criterion. In general, the hardening model has a greater impact on the accuracy of spring back prediction than the yield criterion does.

In the 4S measurement area, the predicted trends of spring back model and the test results are basically the same, but the values are quite different. The maximum position of spring back measured by test is point 21, and the maximum value is 3.49 mm. The maximum positions of spring back obtained by the four prediction models are all at point 14. The maximum values are 5.3 mm, 5.5 mm, 4.8 mm, and 4.0 mm, respectively. The experimental value of spring back at point 14 is 1.21 mm, which is closest to the prediction result of Barlat’89 + Yoshida–Uemori mixed hardening model. However, the prediction values of four spring back models at point 21 are 2.8 mm, 2.7 mm, 2.5 mm, and 2.5 mm, respectively, which is closest to the prediction of Barlat’89 + Hollomon isotropic hardening model. The predicted values of spring back models in other measurement areas are in good agreement with the test values, and the prediction accuracy of Barlat’89 + Yoshida–Uemori mixed hardening model is the highest.

### 4.2. Effect of Hydraulic Pressure

**Figure 8:**
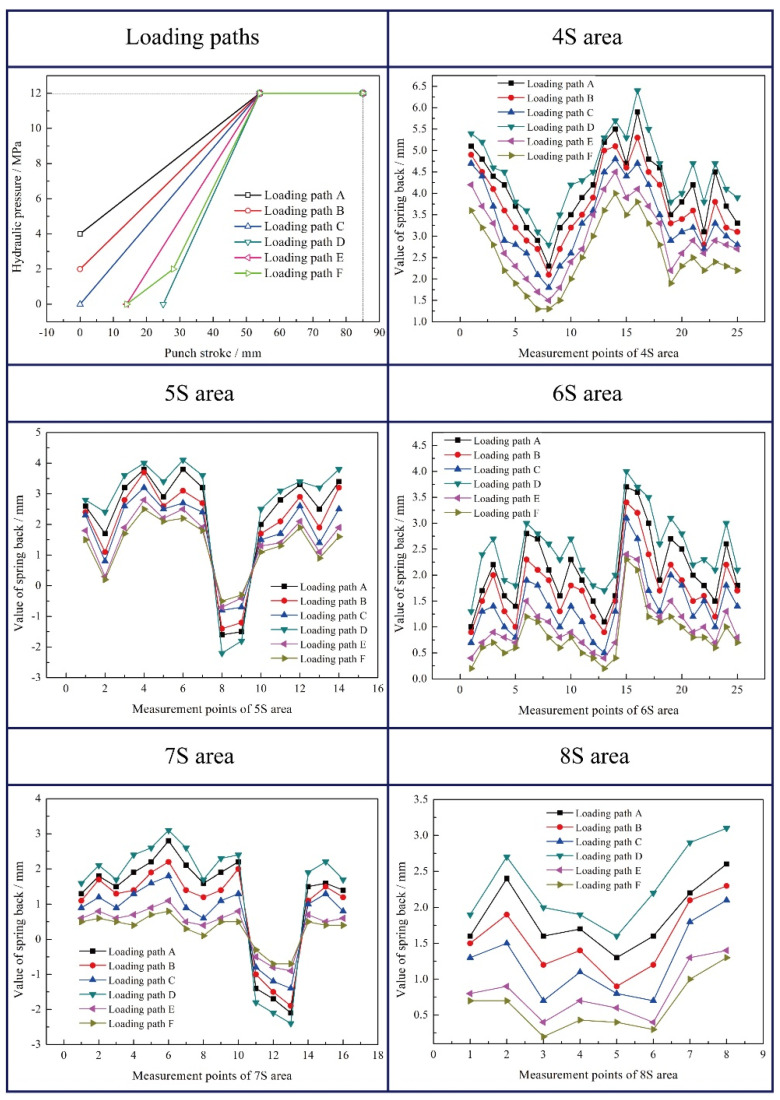
Initial hydraulic pressure paths and the corresponding spring back values of different areas. This figure is the spring back values of different areas (4S–8S areas) under different hydraulic pressure paths. The hydraulic pressure paths are in the first figure of Figure 8.

The Barlat’89 + Yoshida–Uemori mixed hardening model was used to investigate the effect of hydraulic pressure on the spring back behavior of the part due to the fact that it has high accuracy for describing the deformation of aluminum alloy and the process of rigid-flexible sequential loading forming. The initial hydraulic pressure paths designed according to the analysis of Section 2.2 and the corresponding spring back values of different areas are depicted in Figure 8. It can be seen that the spring back values in different areas using loading path D are the largest. Because of the late loading of hydraulic pressure, more blank inflow led to inadequate deformation and large spring back after cutting. Meanwhile, the spring backs of specimens obtained by loading paths A and B with big initial hydraulic pressure are also large due to the high friction level between the blank and punch in initial hydroforming stage. In comparison, the loading paths E and F have the low spring back values and uniform wall thickness distribution. The suitable loading stroke in paths E and F means the blank will not be intimately attached to the sharp features on the punch and have the uniform flow. Finally, the optimized path F has the smallest spring back and the best result.

**Figure 9:**
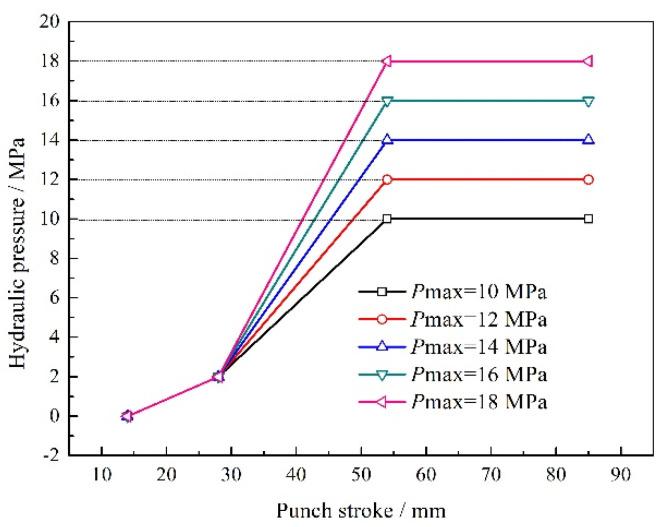
The designed five loading paths of maximum hydraulic pressure.

**Figure 10:**
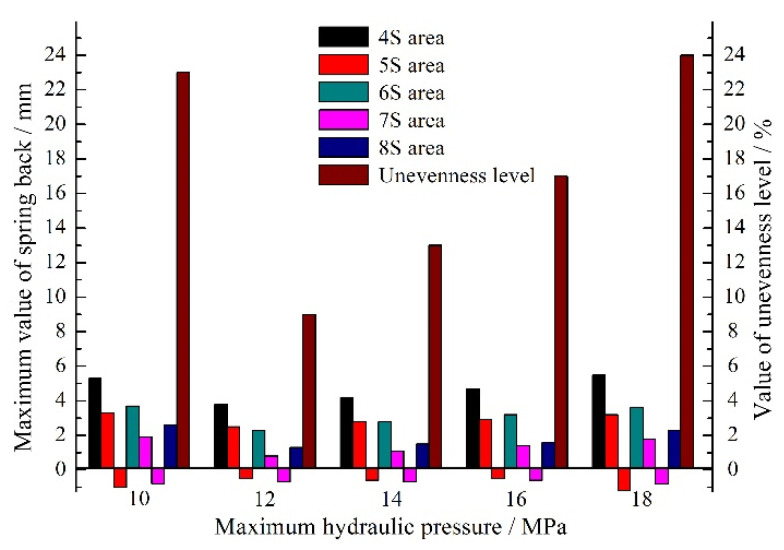
The maximum spring back values and unevenness level of wall thickness with different maximum hydraulic pressures.

For the spring back of the specimen, the maximum hydraulic pressure also plays a significant role. If it is too large, the concave rounded corner of the part will fracture due to the failure of materials filling during the deformation process. If it is too small, the convex rounded corner of the part will rupture due to the insufficient friction conservation effect. Those will have an important impact on spring back at the same time. Therefore, the designed five loading paths of maximum hydraulic pressure are depicted in Figure 9. The corresponding maximum spring back values are shown in Figure 10. Meanwhile, the unevenness level of wall thickness is introduced for further study on the spring back, which is defined as:

(10) $$\gamma =\frac{t_{\max}-t_{\min}}{t_{0}}\times 100\%$$

where *t*_max_ is the maximum wall thickness and *t*_min_ is the minimum wall thickness. The variation of *γ* is depicted in Figure 10.

It can be seen that when the maximum hydraulic pressure is 12 MPa, the maximum spring back value of the specimen is the smallest, which is 3.8 mm, and the uniformity of wall thickness distribution is the highest. When the maximum hydraulic pressure is 14–18 MPa, the spring back of the specimens varies slightly, but the uniformity of wall thickness distribution varies greatly. Similarly, when the maximum hydraulic pressure is 10 MPa, the wall thickness of the specimen decreases seriously and the distribution uniformity is poor due to the insufficient friction conservation effect during the forming process. Therefore, the selection of the maximum hydraulic pressure needs to satisfy the double conditions of spring back and wall thickness distribution.

### 4.3. Spring Back Measurement and Compensation Results

**Figure 11:**
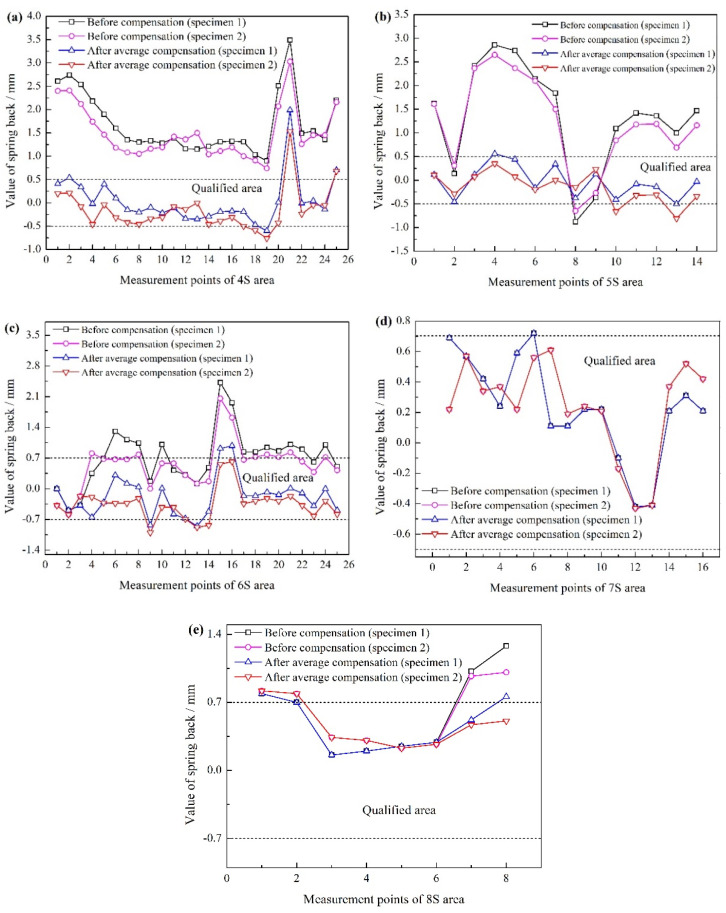
The comparison of spring back values of specimens after compensation: (**a**) 4S area, (**b**) 5S area, (**c**) 6S area, (**d**) 7S area, and (**e**) 8S area.

The comparison of spring back values of specimens after compensation is shown in Figure 11. Before the spring back compensation, the qualified rate of the two measured specimens is 37.5% and 40.91%, respectively. The consistency of spring back values of each measuring point is good, which shows that the measurement accuracy is high. Meanwhile, it can be seen from the figures that the qualified rate of spring back in 4S area is 0%, and the maximum value of spring back is 3.49 mm. The qualified rate in 5S area is 14.29%, and the maximum value is 2.86 mm. The results of spring back measurement of two specimens in 6S area are slightly different. The qualified rate of specimen 1 is 48%, and that of specimen 2 is 64%, the maximum value of spring back of both specimens is 2.42 mm. The qualified rate in 7S area is higher, close to 100%. The spring back value in one place of specimen 1 is only 0.72 mm, which just exceeds the tolerance requirement. The two specimens in 8S area have good consistency, the qualified rate of spring back is 50%, and the maximum value is 1.28 mm. From the above measurement results, it can be seen that the qualified rate of spring back around the part edge is low and the spring back values of the profile are large. The qualified rate of the edge of inner hole and the key checked profile is high, and the corresponding spring back values are small, which are inseparable from the uniform load of high-pressure liquid in the forming process.

The qualified rates of spring back of the two measured specimens after the average compensation through the predicted and measured results are 86.36% and 87.5%, respectively, which are 48.86% and 46.59% higher than those before the spring back compensation. The maximum spring back value is also located in the 4S area, which is 1.99 mm and 42.98% lower than that before the compensation. It is indicated that the suitable spring back compensation using the average compensation method can effectively reduce the spring back of specimens and improve the final forming accuracy for the rigid-flexible sequential loading forming process of aluminum alloy inner panel.

## 5. Conclusions

(1) For the large, complex, multi-feature inner panel, four spring back models are studied, including Barlat’89 + Hollomon isotropic hardening model, Hill’48 + Hollomon isotropic hardening model, Hill’48 + Yoshida–Uemori mixed hardening model, and Barlat’89 + Yoshida–Uemori mixed hardening model. Results show that the hardening model has a greater impact on the accuracy of spring back prediction than the yield criterion does, and the prediction accuracy of Barlat’89 + Yoshida–Uemori mixed hardening model is the highest.
(2) The hydraulic pressure loading path plays a significant role in the specimen spring back. The optimized path F obtains the smallest spring back and uniform wall thickness distribution. Meanwhile, when the maximum hydraulic pressure is 12 MPa, the maximum spring back value of the specimen is the smallest, which is 3.8 mm, and the uniformity of wall thickness distribution is the highest.
(3) The qualified rates of spring back of the measured specimens after the average compensation through the predicted and measured results are improved significantly. The suitable spring back compensation can effectively reduce the spring back of specimens and improve the final forming accuracy for the rigid-flexible sequential loading forming process of aluminum alloy inner panel.

## Figures and Tables

**Table 1:** Mechanical properties of aluminum alloy 5182-O.

Parameters	Value
Yield strength, $\sigma_{s0}$/MPa	119
Yield strength, $\sigma_{s45}$/MPa	118
Yield strength, $\sigma_{s90}$/MPa	116
Tensile strength, $\sigma_{b0}$/MPa	271
Tensile strength, $\sigma_{b45}$/MPa	266
Tensile strength, $\sigma_{b90}$/MPa	264
Elongation rate, $\delta_{0}$/%	28
Elongation rate, $\delta_{45}$/$\delta_{90}$/%	28.5
Work-hardening exponent, *n*	0.34
Anisotropy coefficient, *r*_0_	0.73
Anisotropy coefficient, *r*_45_	0.65
Anisotropy coefficient, *r*_90_	0.6

**Table 2:** Parameters for the revised Yoshida–Uemori hardening model of aluminum alloy 5182-O.

*Y*/MPa	*B*/MPa	*C*	*R*_sat_/MPa	*b*/MPa	*m*	*h*	*C*_1_	*C*_2_
135.62	149.523	432.685	172.853	40.271	10.8	0.25	0.036	0.38

## Data Availability

The data in this manuscript cannot be shared at this time as the data also forms part of an ongoing study.
